# Ophthalmologic and serologic predictors of positive minor salivary gland biopsy in suspected Sjögren’s disease

**DOI:** 10.1038/s41598-025-03284-1

**Published:** 2025-05-28

**Authors:** I. Merve Uçar Baytaroğlu, Ata Baytaroğlu, Suzan Doğruya, Melis Kılıç

**Affiliations:** 1 Rheumatology Clinic, Uşak Training and Research Hospital, Uşak, Turkey; 2 Ophthalmology Department, Hacettepe University, Ankara, Turkey; 3 Ophthalmology Department, Uşak University, Uşak, Turkey; 4 Pathology Department, Uşak Training and Research Hospital, Uşak, Turkey

**Keywords:** Sjögren’s disease, Minor salivary gland, Biopsy, Dry eye, Meibomian, Rheumatology, Eye diseases, Rheumatic diseases

## Abstract

To examine the relation between histopathology, ANA subtypes, and high-resolution dry eye analysis in treatment-naive patients evaluated for Sjögren’s disease suspicion in a referral center. Between October 2022 and December 2023, 231 patients who underwent minor salivary gland biopsies were included in the study. Patients were grouped according to their Chisholm and Mason (CM) biopsy scores. Non-invasive ocular surface evaluations and ANA subtype analysis, according to the International Consensus on ANA Patterns (ICAP), were also conducted. Meibomian gland (MG) atrophy was significantly higher (*p* 0.02) in the positive biopsy group (OR 1.02), although it was a weaker predictor than other parameters. Anti-SSA positivity was markedly correlated with positive biopsy results. However, subtype analysis did not show any significant correlation. In predicting CM scores > 2, ANA positivity was 2.1-fold, SSA positivity was 3.6-fold, Schirmer positivity was 12, fivefold, positive ocular OSS scores were fourfold, limbal redness, and NiBUT less than 10 s were 2.4-fold more significant determinant factors. Our results confirm that the Schirmer test and classical serologic markers are still irreplaceable. We believe that novel measurement techniques that enable faster, non-invasive analyses with advanced technology will facilitate the differential diagnosis of Sjögren’s disease in patients with dry eyes.

## Introduction

Diagnosing Sjögren’s disease is a complex process that involves multiple medical fields. This requires a detailed clinical examination of the eyes and histopathological and rheumatologic evaluations.

The two hallmarks of this disease are xerophthalmia and xerostomia. One of the characteristic features of Sjögren’s disease is glandular dysfunction and atrophy due to lymphocytic infiltration, which primarily affects the lacrimal and salivary glands. Meibomian glands, which are holocrine sebaceous glands located in the upper and lower eyelid tarsus, may also exhibit dysfunction and atrophy, likely secondary to chronic inflammation rather than direct lymphocytic infiltration. According to current literature, SD is defined as secondary SD only if it is accompanied by another concurrent or previously diagnosed autoimmune disease. However, no long-term studies have examined the conversion from primary to secondary. Given that SD has a wide range of clinical phenotypes and can affect multiple organs, its diagnosis and management are complex^1^.

According to the current ACR/EULAR 2016 classification criteria, a positive biopsy result (Chisholm and Mason (CM) grading 3–4) or positive anti-SSA/Ro result and a positive Schirmer test result or ocular staining score can confirm the diagnosis^2^. The negative predictive value of biopsy is reported to be 95% and above, and the positive predictive value can be as high as 63%, even when strict focus-scoring recommendations are considered^3^. It has also been reported that in 20% of primary Sjögren’s cases, the observed focus score can be negative on biopsy, and anti-SSA/Ro positivity may be more valuable, especially in these cases^4^. The literature also includes a comprehensive study of clinicopathological correlations. It has been reported that the Chisolm and Mason scoring methods, which pathologists frequently prefer, and the clinical evaluations of rheumatologists are highly parallel^5^. However, in ophthalmology studies, many guidelines have emphasized that there is a severe discrepancy between patient symptoms and findings, except for quantitative ocular surface staining and Schirmer scores^6,7^. Furthermore, both primary (pSD) and secondary Sjögren’s disease have been shown to lead to aqueous insufficiency, meibomian gland disease, and associated evaporative dry eye^8^. Since ocular tear film components are secreted from the lacrimal and meibomian glands, as well as the accessory lacrimal apparatus and other glands, meibomian analysis is a valuable diagnostic tool in these cases. Salivary gland scintigraphy is another novel diagnostic tool for SD, especially in cases in which histopathologic and serologic criteria do not overlap^9^. However, more non-invasive supportive tests with high patient compliance are still needed for in-between cases.

In summary, the main challenges in diagnosing primary and secondary SD are a heterogeneous presentation with potential overlap with other autoimmune diseases, a lack of serologic, histopathologic, or ophthalmic early biomarkers, variability in histopathologic findings, and interobserver variability in minor salivary gland biopsies, and the fact that serologic tests (anti-SSA and anti-SSB) do not exclude the diagnosis^5,10–13^. While conventional ocular parameters are frequently used in SD studies in the literature; similarly, the complete ANA profile is often not examined. The possibility that the ocular surface disease index (OSDI) and Schirmer scores are highly affected by patient and practitioner bias in ocular evaluation also limits the reproducibility of these measurements. Therefore, this study aimed to investigate the relationship between histopathologic findings, ANA antibody subtypes, and dry eye analysis data in patients who were evaluated for suspected SD in our ophthalmology and rheumatology clinic and who had not been previously treated for rheumatic disease or ocular surface disease.

## Method

In this cross-sectional study two hundred and thirty-one patients between the age of 18–75 were evaluated in our ophthalmology and rheumatology clinic with suspected Sjögren’s disease. All cases were recently evaluated for symptoms or signs that appeared within the last 6 months or were referred to us for diagnostic confirmation with clinical suspicion within this period. All patients underwent thorough ophthalmic examination; in addition to the ocular surface disease index questionnaire (OSDI), anterior segment evaluation consisted of biomicroscopic evaluation, Schirmers’s test, and ocular staining score (0–12) was calculated by cornea staining with preservative-free 1% fluorescein and conjunctival staining with lissamine green staining strips, and high-resolution dry eye analysis with Sirius + (Costruzione Strumenti Oftalmici, Firenze, Italy) embedded software Phoenix v.4.1.3.1. Subjects with a history of any chronic ocular disease requiring longstanding use of topical medication (e.g., glaucoma), contact lens wear, or any corneal (e.g., refractive surgery) or intraocular surgery were excluded. In symptomatic cases with low ocular staining scores or negative Schirmer’s test results, systemic or musculoskeletal complaints were sought to indicate rheumatological evaluation.

In the rheumatologic examination, in addition to a detailed physical examination, complete blood count, ESR, CRP, ANA, and ENA panel, RF, and CCP laboratory analyses were performed, taking into account the patients’ signs and symptoms. A minor salivary gland biopsy was performed in all cases necessary for diagnostic or confirmatory purposes. Biopsies were performed with an approximately 1 cm incision with a no.11 blade in accordance with the EULAR guidelines^14^.

Patients who underwent initial rheumatologic evaluation were referred to A.B. for ophthalmic evaluation, followed by anterior segment imaging and device-dependent measurements by S.D. in the same week, masking the initial examination findings. All pathology preparations were evaluated by M.Y., blinded to the physical examination and laboratory findings.

**Fig. 1:**
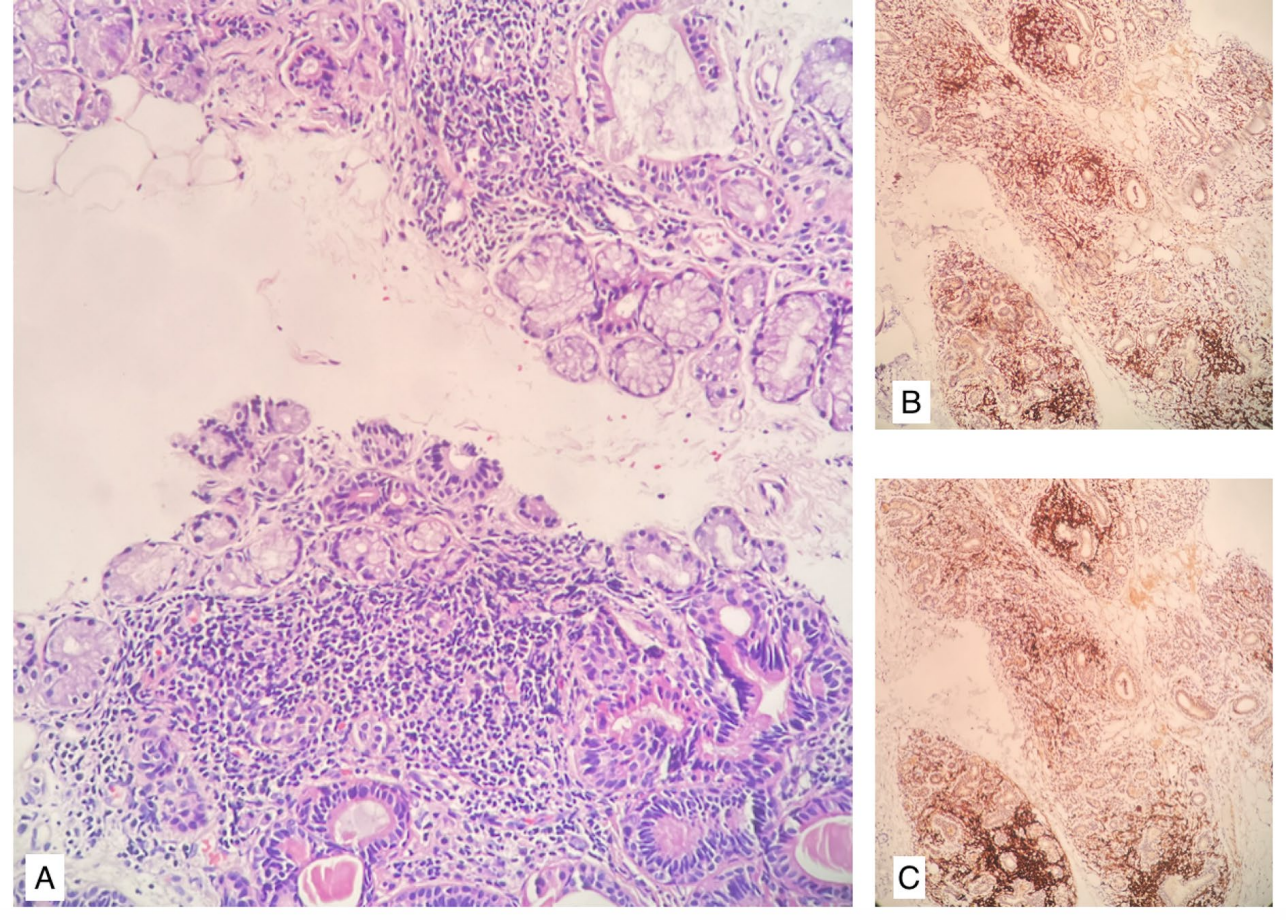
Minor salivary gland with several lymphocytic foci, consistent with CM 4. (**A**) Hematoxylin and eosin (H & E) stain. (**B**) CD3 and (**C**) CD20 stains highlight lymphocytic foci.

The biopsies were scored according to the Chisholm and Mason scoring system (CM). For this purpose, 4 µm thick hematoxylin and eosin-stained sections were taken from formalin-fixed paraffin-embedded blocks and examined by pathologists. At least 6 sections were examined for each biopsy, the areas of the biopsy sections were measured, and the salivary gland lobules were counted. Biopsies with an area of at least 4 mm^2^ and containing 4 salivary gland lobules were considered sufficient for evaluation. The lymphocytic focus is defined as an aggregate of at least 50 mononuclear cells. The focus score (FS) represents the number of lymphocytic foci observed in every 4 mm^2^ area. To determine the FS, lymphocytic foci were counted separately in each section and the focus number was divided by the surface area and multiplied by 4. The section with the highest FS was used to determine the CM degree. CM scoring categorizes this glandular infiltration in 4 degrees. Grade 0 is a healthy tissue with the absence of any lymphocytic infiltration (0 foci per 4 mm^2^). Grades 1 and 2 indicate mild to moderate infiltration that does not qualify as a focus score. A single focus (1 focus per 4 mm^2^) is reported as grade 3, and the presence of more foci per 4 mm^2^ is reported as grade 4 (Fig. 1)^2,5,15^. The Chisholm and Mason scoring system (CM) is a pivotal method for evaluating minor salivary gland biopsies, particularly for diagnosing SS. This scoring system classifies the degree of lymphocytic infiltration in glandular tissue, which is a critical histopathological feature for confirming SS. According to established criteria, a focus score (FS) of ≥ 1 focus per 4 mm^2^ of glandular tissue indicates significant lymphocytic infiltration, which is essential for a positive diagnosis of SS. Throughout the text, the patients with CM scores of 3–4 were referred to as Group 1, and those with lower scores were referred to as Group 2.

SPSS 15.0 for Windows was used for statistical analysis. When the normal distribution condition was met, descriptive statistics were presented as numbers and percentages for the categorical variables. For numerical variables, mean, standard deviation, minimum, and maximum were given if the normal distribution condition was met and if no median interquartile range was given for numerical variables. The ratios given as percentages in the tables for the groups were compared using the Chi-Square Test. Comparisons of numerical variables in two independent groups were made with the Student t-test when the normal distribution condition was met (values indicated as min–max in the tables) and the Mann–Whitney U test when the normal distribution condition was not met (values indicated as median in the tables). The parameters evaluated to predict clinically significant biopsy results (Chisolm 3–4) were analyzed using univariate logistic regression. The presence of statistically significant determinant factors was further examined using Logistic Regression Analysis, in which the effect of factors with P < 0.250 in univariate analyses was evaluated together according to age and sex. The alpha significance level was accepted at p˂0.05.

The study protocol was approved by the Ethics Committee of the Uşak University Faculty of Medicine (approval number: 235-235-02). All procedures were performed in accordance with the ethical standards of the National Research Committee, the 1964 Helsinki Declaration and its later amendments, and comparable ethical standards. Written informed consent was obtained from all the participants.

## Results

Between October 2022 and December 2023, 231 patients aged 18–75 years who underwent minor salivary gland biopsy with suspected Sjögren’s disease because of clinical and laboratory findings were included in the study. Of the patients included in this study, 203 were female. When grouped in terms of biopsy scores, there was no significant difference in sex distribution between the groups (*p* 0.4). The mean patient age was 55 ± 12 years. There was no significant difference between the groups in terms of the age distribution (*p* 0.63). When the distribution of the biopsy results was analyzed, no lymphocytic infiltration was detected in 53 cases, and the CM score was reported as 0; the CM score was 1 in 93 cases and 2 in 20 cases. The number of cases with CM scores of 3 and 4 was 37 and 28, respectively. The rate of RF positivity observed in all cases was 23.4%, and the rate of ANA positivity detected without titration or grading was 64.1%. The demographic and CM score characteristics of the patients are summarized in Table 1. The ANA and Schirmer positivity rates were significantly higher in group 1 than in group 2 (*p* 0.025, *p* < 0.001). When the OSS scores of the groups were compared, a statistically significant difference was observed (*p* 0.003). There was also a statistically significant difference in the limbal redness levels between the groups (*p* < 0.001). While the rate of meibomian gland (MG) atrophy was significantly higher in group 1, the Non-invasive break-up time NiBUT(sec) was calculated as a median of 6.4 (4.1–9.8) s in the first group and 10 (5.6 -12) seconds in group 2 (*p* 0.020, *p* 0.001). When 10 s was taken as the threshold value in the NiBUT measurements of the groups, statistical significance was maintained (*p* 0.011). The SSA positivity rate of patients in the first group was also significantly higher than that of patients with lower CM scores in group 2 (*p* < 0.001). In terms of the OSDI scores, in which the patients rated their complaints subjectively, the median value was 27, and there was no significant difference between the two groups (*p* 0.38). No significant difference was observed in the calculation, in which a value of 33 was taken as the threshold for all patients (*p* 0.18). Although tear film layer thickness was measured as a median of 180um in group 1 and a median of 215 um in group 2, there was no significant difference between the groups (*p* 0.135).

**Table 1:** Distribution of cases according to demographic data and biopsy results.

		Total	Biopsy results (CM)		*p*
			CM 3–4	CM 0–2	
Age (Min–Max)		55.1 (21-80)	55.7 (30-80)	54.8 (21-78)	0.629
Sex n (%)	Female	203 (87.9)	59 (90.8)	144 (86.7)	0.400
	Male	28 (12.1)	6 (9.2)	22 (13.3)	
RF n (%)	Negative	177 (76.6)	47 (72.3)	130 (78.3)	0.332
	Positive	54 (23.4)	18 (27.7)	36 (21.7)	
ANA n (%)	Negative	83 (35.9)	16 (24.6)	67 (40.4)	0.025**
	Positive	148 (64.1)	49 (75.4)	99 (59.6)	
Anti-SSA n (%)		57 (24.7)	28 (43.1)	29 (17.5)	< 0.001**
AC-1 n (%)		13 (5.6)	2 (3.1)	11 (6.6)	0.361
AC-2 n (%)		29 (12.6)	6 (9.2)	23 (13.9)	0.386
AC-3 n (%)		8 (3.5)	2 (3.1)	6 (3.6)	1.000
AC-4 n (%)		61 (26.4)	18 (27.7)	43 (25.9)	0.782
AC-5 n (%)		7 (3.0)	1 (1.5)	6 (3.6)	0.676
AC-8/9/10 n (%)		12 (5.2)	4 (6.2)	8 (4.8)	0.744
AC-13 n (%)		3 (1.3)	2 (3.1)	1 (0.6)	0.192
Schirmer < 5 mm/5 min n (%)	Negative	170 (73.6)	24 (36.9)	146 (88.0)	< 0.001**
	Positive	61 (26.4)	41 (63.1)	20 (12.0)	
Ocular staining score n (%)	< 5	192 (83.1)	45 (69.3)	147 (88.6)	0.003**
	≥ 5	39 (16.9)	20 (30.8)	19 (11.4)	
OSDI score median (IQR)		27 (16–38)	28 (19.5–42)	27 (16–36)	0.384
OSDI score n (%)	< 33	147 (63.6)	37 (56.9)	110 (66.3)	0.184
	≥ 33	84 (36.4)	28 (43.1)	56 (33.7)	
Limbal redness (0–4)	0	99 (42.9)	17 (26.2)	82 (49.4)	< 0.001**
	1	88 (38.1)	29 (44.6)	59 (35.5)	
	2	38 (16.5)	14 (21.5)	24 (14.5)	
	3	5 (2.2)	5 (7.7)	0 (0.0)	
	4	1 (0.4)	0 (0.0)	1 (0.6)	
Limbal redness Median (IQR)		1 (0-1)	1 (0-2)	1 (0-1)	< 0.001**
Meibomian atrophy ratio Median (IQR)		26 (15-38)	32 (20.5-44)	25 (14-34.1)	0.020**
Tear film layer thickness (um) Median (IQR)		210 (140-290)	180 (130-285)	215 (160-290)	0.135
NiBUT (sec) Median (IQR)		8.4 (4.7-12)	6.4 (4.1-9.75)	10 (5.55-12)	0.001**
NiBUT (sec) n (%)	< 10 s	156 (67.5)	52 (80.0)	104 (62.7)	0.011**
	≥ 10 s	75 (32.5)	13 (20.0)	62 (37.3)	

In univariate logistic regression analysis examining the effect of the parameters evaluated in predicting CM scores > 2, ANA positivity was 2.1-fold, SSA positivity was 3.6-fold, and Schirmer positivity measured in ophthalmologic evaluation was 12.5-fold. Positive OSS scores (≥ 5) were found to be approximately fourfold, limbal redness increased gradually with increasing grades, with an average of 2.4-fold, and NiBUT less than 10 s were found to be 2.4-fold more significant determinant factors. Although the rate of MG atrophy was significantly (*p* 0.02) higher in the group with a higher biopsy score, this increased rate was found to be a weaker determining factor (OR 1.02) compared to other parameters. Age, sex, RF positivity, OSDI score, tear film thickness, and ANA subgroups were not associated with positive biopsy results. Further analysis, in which the effects of the factors with *p* < 0.250 in univariate analyses were evaluated together according to age and sex, revealed that ANA positivity was 2.6 times (*p* 0.026), Schirmer positivity was 12.8 times (*p* < 0.001), and SSA positivity was 4.6 times (*p* < 0.001) statistically significant determinants. Logistic regression analysis of factors associated with a significantly positive biopsy result is summarized in Table 2.

**Table 2:** Univariate logistic regression analysis of parameters in predicting a positive biopsy result.

	*p*	OR	95% C.I	
ANA (Ref: Negative)	0.026**	2.630	1.121	6.173
Schirmer test (Ref: Negative)	< 0.001**	12.789	5.501	29.735
OSS (Ref:0)	0.868			
< 5	0.598	0.789	0.326	1.907
≥ 5	0.802	1.174	0.336	4.094
OSDI Score (Ref: < 33) ≥ 33	0.363	0.679	0.295	1.564
Limbal redness (Ref:0)	0.887			
Grade 1	0.290	1.602	0.669	3.836
Grade 2	0.506	1.515	0.446	5.146
Grade 3	0.999			
Grade 4	1.000			
Meibomian atrophy ratio	0.853	1.003	0.976	1.030
NiBUT sec (Ref: > 10 s)	0.355	0.639	0.247	1.653
Anti-SSA (Ref: Negative)	< 0.001**	4.563	1.991	10.454
AC-1 (Ref: Negative)	0.304	0.447	0.096	2.076
AC-2 (Ref: Negative)	0.343	0.632	0.245	1.632
AC-3 (Ref: Negative)	0.841	0.847	0.166	4.306
AC-4 (Ref: Negative)	0.782	1.095	0.575	2.088
AC-5 (Ref: Negative)	0.422	0.417	0.049	3.530
AC-8/9/10 (Ref: Negative)	0.682	1.295	0.376	4.458
AC-13 (Ref: Negative)	0.179	5.238	0.467	58.786
Sex (Ref: Male)	0.969	1.025	0.287	3.658
Age	0.547	0.990	0.956	1.024

Statistical significance was accepted as <0.05, and these values are shown in asterisks.

**Fig. 2:**
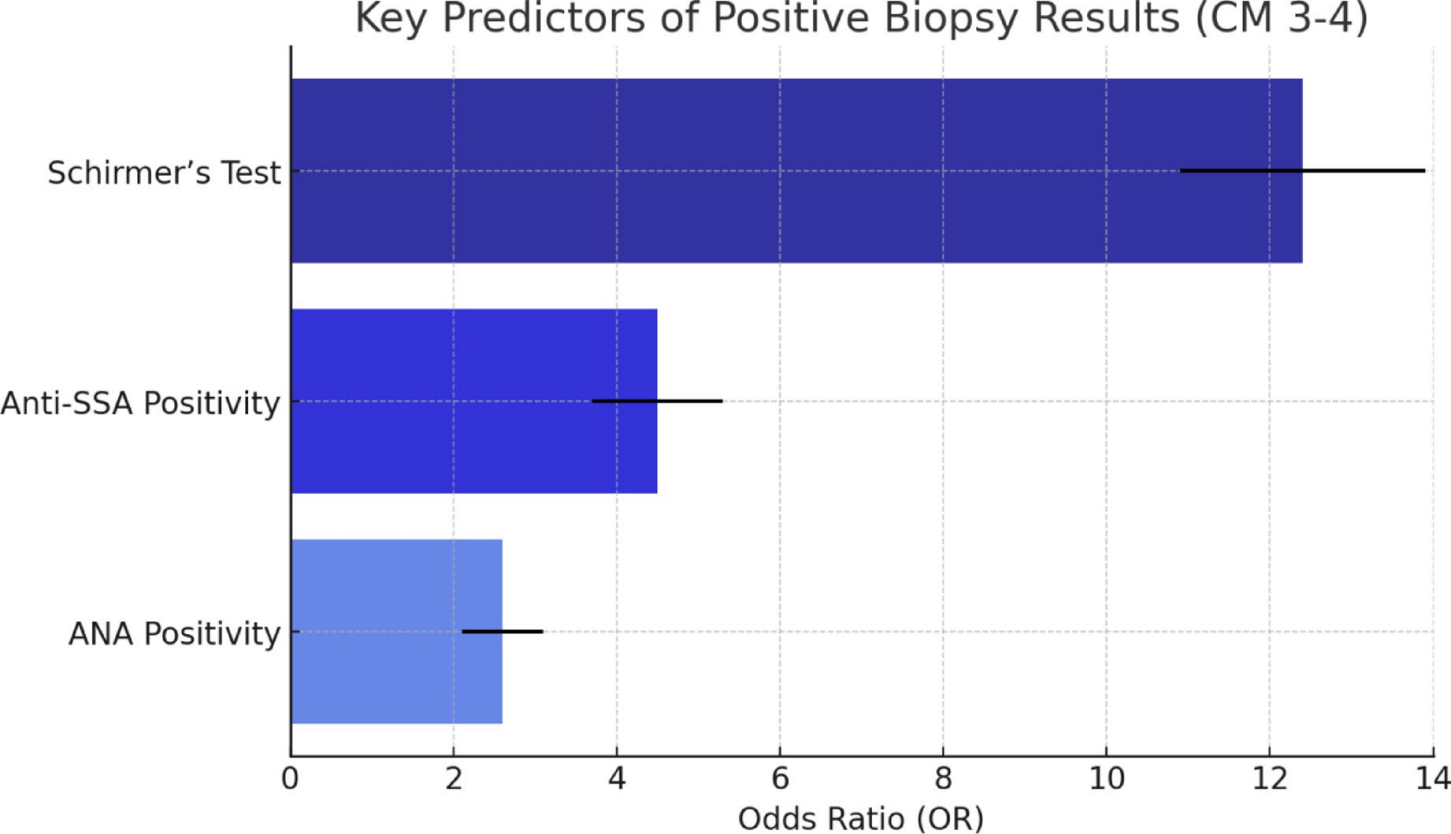
Bar chart summarizing the key predictors of positive biopsy results (CM 3–4), showing their odds ratios (OR).

In summary, according to our study results, when we accept histopathologic evaluation as the gold standard, the most significant association (in terms of OR) with positive biopsy results in cases evaluated with suspicion of SD is as follows (Fig. 2):

- Positive Schirmer’s test—12.4-fold
- Anti-SSA positivity—4.5-fold
- ANA positivity regardless of subtype—2.6-fold
- Although the rates of limbal redness, ocular surface staining, and atrophy of eyelid meibomian glands were found to be significantly different between histopathological groups, regression analysis according to age and sex revealed that their effects were not statistically significant.

## Discussion

In a more extensive series of biopsy studies conducted in our country, cases with similar demographic characteristics were grouped as SD or non-SD according to their diagnoses, and histopathologic correlation was examined for laboratory findings and histomorphological findings, such as ductal dilatation and acinar atrophy. In this study, Schirmer test scores were analyzed in three categories, and scores < 5 mm were considered positive and accepted as dry eye without any additional ophthalmologic evaluation. Throughout the study, it was emphasized that a CM score of ≥ 3 was the most critical clinical determining factor^15^. Vivas et al. retrospectively analyzed approximately 1000 biopsies and reported that in approximately 260 patients diagnosed with SD, clinical and pathologically confirmed diagnoses were frequently compatible. They also reported SSA positivity in only 15% compared to our finding of 25%, ANA positivity in 41% compared to our 64%, and RF positivity in 13% of their biopsy-positive cases compared to our result of 23%^5^. Notably, no detailed ophthalmologic analysis was performed in this extensive series of studies, and dry eye evaluation was only reduced to the Schirmer test result. Considering that the diagnoses may change and the concordance between biopsies is low, especially in cases with repeat biopsies after an average of 5 years, it is clear that parameters other than serological markers and biopsy findings should be examined^16^. The relatively higher rates of serological positivity in our study can be explained by a referral following a more thorough ophthalmologic evaluation with a higher level of clinical suspicion. Considering that the incidence and prevalence of pSD in the community are 0.004% and 0.1–0.60%, respectively, the fact that only 65 of the patients referred during the 15 months of our study had positive results for minor salivary gland biopsy would be an overestimated number in terms of pSD. In light of the current data, the secondary conversion rate of pSD cases was reported to be 4.4% in approximately 5 years, and the annual incidence was reported as 9/1000^17^. However since almost all of these individuals had not previously undergone a rheumatologic evaluation, we anticipate that almost half of them will be classified as having secondary SD at prospective follow-up. In addition, the current ACR/EULAR-2016 criteria are not diagnostic but classification criteria based on a consensus approach using a data-driven methodology involving a large number of international patient cohorts. There was no difference between pSD and sSD in terms of diagnostic criteria. As they both share similar clinical and laboratory findings as well as similar histopathological features except for perivascular infiltrates in the biopsies, we did not find it necessary to make a primary-secondary distinction in this study in which we examined the correlation between the current diagnostic tests^18,19^.

### Biopsy correlation with ANA results

In a study conducted before the current ACR/EULAR criteria, Akpek et al. showed that 15–20% of newly diagnosed pSD cases were seronegative. This study found ANA positivity in only 1/3 of the newly diagnosed pSD cases^20^. In a similar study, Goel et al. reported that SS was diagnosed in only one of 47 cases despite a negative biopsy result. However, this patient was also seropositive for ANA and RF^21^. In a recent study, 209 pSD cases were analyzed. It was found that 68% of the SS-A positive cases were also biopsy positive, and > 65% of the ANA pattern detected was in a speckled pattern; however, subgroup analysis with the current International Consensus on ANA Patterns (ICAP) nomenclature was not performed in this study^22^. Another difference in the compared study is that the AECG(American-European Consensus Group)-2002 classification was used with a more stringent criteria scoring. In contrast, according to the ACR-2016 classification, a positive Schirmer’s result, dry eye complaints, and a positive biopsy were sufficient for diagnosis. In our study, we used the ACR-2016 criteria to provide diagnostic criteria for a broader patient population. Although anti-SSA and ANA/AC-4 positivity, which were found at similar rates in our study in parallel with the literature, were correlated, anti-SSA was found to be significantly different between the groups in terms of correlation with the biopsy score. In contrast, the AC-4 positivity was not statistically significant. We think that this is since AC-4 includes Mi-2, TIF-1ɣ, and Ku types, which are subclassified as AC-4b apart from SSA and SSB and whose positivity is also common in the average population^23^.

In addition, RF positivity is another common finding in patients with SS; studies have shown that RF can be present in a significant percentage of patients with SD, with estimates ranging from 30% to over 60%^24^. A high rate of RF positivity is strongly associated with extra-glandular manifestations^24,25^. However, since almost all objective and subjective findings evaluated in our study were related to glandular involvement, no correlation with RF was found, as expected.

### Biopsy correlation with high-resolution dry eye analysis and meibomian gland atrophy

In their meibography study using the transillumination method in a limited number of cases, Shimazaki et al. showed that more than 50% of SD cases had more than 50% inferior tarsal MG atrophy. However, we observed much lower ratios of atrophy (32%), even in Group 1. Another noteworthy point in their study was that, although a significant difference was found between SD and non-SD dry eye patients in terms of staining patterns, unlike our results and the current literature, no significant difference was found in terms of Schirmer scores or TBUT results^26^. The fact that no difference was observed between the patient groups in terms of tear film layer thickness may be partially explained by the hypothesis that the inflammatory cycle that occurs in SD creates a thicker but unstable ocular tear layer in a compensatory effort to overcome corneal neuralgia, which may suggest thicker measurements of the corneal tear film layer and NiBUT duration^27^. A study of 108 patients using a different but similar interferometry-based device also found that the severity of MG atrophy was highly correlated with disease duration in patients with pSD^28^. In contrast, MG atrophy can occur in a substantial proportion of the population, with prevalence rates as high as 72% in adults^29^. The underlying cause of atrophy has been hypothesized to be the initial hyperkeratinization of the ducts, leading to obstruction and stasis, thus explaining the higher rates of atrophy observed in elderly patients^30,31^. Although biopsy, serology, and ocular surface changes constitute the three pillars of SD diagnosis, a high correlation between biopsy and ocular surface findings is expected since both indicate glandular involvement. However, it should be emphasized that MG atrophy in SD cases is probably more related to the chronic inflammatory process rather than the primary glandular involvement of the disease. Recent studies have emphasized that MG atrophy in SD cases may be a critical non-invasive biomarker for early diagnosis^32^. However, Schirmer’s test still seems to be the gold standard method for ocular surface and tear assessment in our study, as OSS scores presented similar odds ratios with both limbal redness grading and MG atrophy ratio measured by non-contact methods, even though logistic regression analysis showed no correlation with biopsy results, suggesting that these analyses can be used instead of the OSS and van Bijsterveld scoring methods, which require ocular surface staining with two different dyes. Current literature indicates that patients with SD typically exhibit higher OSDI scores than those with other forms of dry eye disease. Most OSDI scores in patients with SS often fall within the moderate-to-severe range. It has been reported that patients with SD have OSDI scores that are higher than those of other systemic autoimmune diseases, such as systemic sclerosis^33^. In contrast, it was also shown that patients with primary SD had lower OSDI scores than those with age-matched non-Sjögren’s dry eye, suggesting a complex relationship between symptoms and underlying pathology^34^. All of these factors reflect the substantial burden of ocular symptoms associated with the disease. In our study, no significant difference was found in biopsy scores when OSDI scores were evaluated both as continuous numerical variables and categorically as OSDI scores above and below 33. Although these data are in line with the ACR/EULAR-2016 criteria evaluation that anti-SSB positivity and oral and ocular symptoms do not contribute significantly to the classification, we think that the OSDI score should be considered not only as a result of the involvement of lacrimal glandular structures but also as a reflection of pathogenesis in which neuroinflammatory processes affecting corneal sensitization play a role. Unfortunately, there is currently no non-invasive/non-contact methodology to examine the correlation between these two parameters. Hopefully, the increasing trend for novel biomarker identification using advanced bioinformatics and machine learning techniques for SD highlights the potential for more accurate and early diagnosis and the development of targeted therapies. It is noteworthy that continuous advances in the field of ocular surface imaging with AI-based technologies will probably enable the diagnosis and quantification of ocular changes with a higher rate of reliability^35^. Although the importance of kits showing more specific reactivity in terms of ANA subtypes is increasing, no significant change in serological criteria is expected. However, there is still no consensus on the diagnostic value, sensitivity, and specificity of upper- and lower-lid tarsal meibomian gland imaging. The correlation of the different algorithms used by different imaging devices with each other, their reproducibility, and their correlation in larger series should be examined to determine the diagnostic value, sensitivity, and specificity of imaging. In addition, the lack of studies in the literature on the use of non-invasive ocular surface imaging to evaluate treatment efficacy and follow-up provides a good opportunity for new areas of study.

### Limitations

The most important limitation of our study is that it was cross-sectional and based on referred patients, which may have introduced selection bias. This limits the ability to establish a causal relationship between ophthalmologic and rheumatologic findings and Sjögren’s disease progression. Moreover, the study population consisted primarily of patients from a single ophthalmology and rheumatology clinic. Patients with a history of chronic ocular diseases, contact lens wear, or previous ocular surgery were excluded. While this helps maintain a more homogenous sample, it may limit the applicability of our findings to a broader patient population with comorbid conditions. that may not be representative of a broader population. In addition, the cases included in this study were referred to us with clinical suspicion without differentiating between primary or secondary SD and without a thorough assessment of possible underlying rheumatologic involvement. Since masking was applied during measurements and analysis, follow-up data revealing secondary SD cases were not presented in the study. Finally, we considered biopsy results as the primary diagnostic tool; however, it is an invasive procedure and may have interobserver variability in histopathological interpretation. However, some patients with negative biopsy results may still have SD based on the clinical criteria.

## Conclusion

Our study aimed to evaluate the correlation between non-invasive ocular surface analysis and serologic non-anti-SSA ANA subtypes and the results of minor salivary gland biopsies regarding their diagnostic use. We confirmed that the current ACR/EULAR-2016 criteria are crucial for evaluating objective findings. We believe that future non-contact analyses, which have emerged with the development of technology, will replace the current ocular surface tests with graded and more objective and repeatable measurements, especially in evaluating patients with dry eye and suspected Sjögren’s disease.

## Data Availability

Data supporting this study’s findings are available only from the corresponding author upon reasonable request. However, owing to current legal regulations, patients’ pathology results cannot be shared.
